# How history trails and set size influence detection of hostile intentions

**DOI:** 10.1186/s41235-022-00395-5

**Published:** 2022-05-12

**Authors:** Colleen E. Patton, Christopher D. Wickens, Benjamin A. Clegg, Kayla M. Noble, C. A. P. Smith

**Affiliations:** Colorado State University, Fort Collins, USA

**Keywords:** Visual search, Intent perception, Visual attention, Uncertainty

## Abstract

Previous research suggests people struggle to detect a series of movements that might imply hostile intentions of a vessel, yet this ability is crucial in many real world Naval scenarios. To investigate possible mechanisms for improving performance, participants engaged in a simple, simulated ship movement task. One of two hostile behaviors were present in one of the vessels: Shadowing—mirroring the participant’s vessel’s movements; and Hunting—closing in on the participant’s vessel. In the first experiment, history trails, showing the previous nine positions of each ship connected by a line, were introduced as a potential diagnostic aid. In a second experiment, the number of computer-controlled ships on the screen also varied. Smaller set size improved detection performance. History trails also consistently improved detection performance for both behaviors, although still falling well short of optimal, even with the smaller set size. These findings suggest that working memory plays a critical role in performance on this dynamic decision making task, and the constraints of working memory capacity can be decreased through a simple visual aid and an overall reduction in the number of objects being tracked. The implications for the detection of hostile intentions are discussed.

## Introduction

The ability to detect hostile behavior is an important demand for human cognition. In military settings, accurately and rapidly detecting hostile behavior can mean life or death. For example, timely identification of the potential threat from an incoming vessel or plane among all the other traffic in maritime environments is crucial to be able to take necessary countermeasures. While current technology might offer an abundance of useful information about nearby ships for identifying potential threats, it is not always accurate or available, especially for entities at far distances (Dahbom & Nordlund, 2013). Specific cues surrounding movement, such as the course of a ship or coordinated activities, can raise suspicions of hostility in ships (Lane et al., 2010; Liebhaber et al., 2002). This may be particularly useful when technologies that gather information on hostility, such as hull numbers or port destinations, fail or are unavailable. The question then arises as to how hostility might be expressed and detected in geospatial behavior, such as movement of ships, at a distance beyond visual range as represented on a radar or GPS display (Riveiro et al., 2018).

To investigate this issue, Patton et al. (2021) used a basic simulation of an open water maritime environment. This research highlighted the difficulty in detection of hostile intent from spatial movements alone, particularly where any level of noise in the movements served to mask the other purposeful actions. Two hostile behaviors were investigated: *hunting*, where the hostile ship moves continuously closer to the usership until eventually reaching it; and *shadowing*, where the hostile ship stalks the usership at a constant distance by performing the same movements. Several distractor (non-hostile) ships were also moving, and people had to identify both which ship was hostile and the hostile behavior exhibited. The movement of the hostile target was also perturbed by occasional noise that partially masked its purposeful hostile behavior. The current study employed a variant of the Patton et al. (2021) platform to explore what elements impact hostile intention detection, and hence where there may be key opportunities to support a human operator in this demanding task. Insights into the ShadowHunt paradigm with its dynamic, iterative spatial demands might be gleaned from links to four existing paradigms or subtasks in perceptual/cognitive psychology. These include:

### Multi-object tracking (MOT)

In the MOT paradigm, the observer is shown one or more dynamic entities (targets) of which to “keep track” amidst several non-targets. After monitoring for a period in which all entities are moving, they must respond to a probe of one of the entities, and their performance is assessed by the accuracy with which they classify a probe correctly as one of the assigned targets (Pylyshyn & Storm, 1988; Bettencourt & Somers, 2009; Yantis, 1992; Schooner et al., 2020; Mackenzie et al., 2017, 2021; see Meyerhoff et al., 2017 for a comprehensive review).

The ShadowHunt paradigm invokes the necessity of visually tracking multiple objects as there are multiple ships on the screen of unknown intentions that must be watched. However, the main difference is that in MOT paradigms, people are told in advance which are the target items and must remember them; but in the current paradigm they must search for and identify the target and also diagnose its form of behavior. Additionally, ShadowHunt requires choices about the movement of the usership which in turn elicit different cues about target identity, and no such features are present in the typical MOT task (for some exceptions, see *Contingent Behavior* section below).

Although there are clear differences in the paradigms, several variables often examined in the MOT paradigm may also be relevant to the current research. For instance, it is known that the increased separation of targets makes detection more difficult both in MOT studies and, specifically, the current paradigm (Shim et al., 2008; Patton et al., 2021). Additionally, MOT studies have shown that increasing the number of objects increases working memory demands, which reduces performance (for review, see Meyerhoff et al., 2017). We expect that pattern to emerge in the current study as well, due to the similarity in object tracking between paradigms.

### Visual search

The literature on applied visual search is vast (see Wickens & McCarley, 2008; Wickens et al.,(2022), for a summary), and search, in this case for the single hostile element, is a critical component of the ShadowHunt paradigm. A small number of studies have combined visual search and MOT, examining the interference between the two (e.g., Gao et al., 2019; Thornton et al., 2021). This combination of the two subtasks is embodied in the “wolves and sheep” paradigm (Gao et al., 2009; Gao and Schol 2011, 2019) in which multiple moving elements are displayed, one of which is a hostile “wolf” that is stalking or approaching (i.e., hunting) the participant’s own icon or avatar. The results generally show the profound degrading effect of noise or random trajectory perturbations on the element’s trajectories (Gao and Schol 2011, 2019) as well as of number of elements (Gao et al., 2019). Indeed detection of a hostile element in noise falls below 80% accuracy even when the number of objects to be tracked is low (Gao et al., 2019). The former effect has also been revealed in the current paradigm such that detection was increasingly hindered by greater degrees of randomness in the paths of the entities present (Patton et al., 2021).

#### Contingent behavior

One version of the wolves and sheep paradigm examined by Gao and Scholl (2011) required participants to control their own avatar and hence, as in ShadowHunt, to elicit behavior from the hostile target. Although Gao and Scholl (2011) focused on different task goals than ShadowHunt—avoiding the hostile target as compared to diagnosing the behavior of a hostile ship—we expect, given their findings, the large detrimental effect of set size to be seen in the current study as well.

### Dynamic decision making

The ShadowHunt paradigm shares many general properties with dynamic decision making paradigms (Edwards, 1962; Kersthold & Raaijmakers, 1997; Gonzalez et al., 2017) in which diagnostic evidence is accumulated through a series of observations of feedback from participant decisions. In ShadowHunt, this evidence is acquired from the contingent movement of the pair of objects: usership and the suspected target, in order to both infer which is the hostile ship, and diagnose its type of hostility. In a review of the dynamic decision making literature, Kersthold and Raaijmakers (1997) note the poor accuracy of operators in most dynamic decision tasks. They list three possible reasons for this: capacity limitations, lack of knowledge for the decision problem, and a mismatch of the problem and the mental model. Performance in a previous version of ShadowHunt (Patton et al., 2021) hovered around 60%, well above chance but also far short of optimal performance. In this context, a likely candidate explanation for relatively poor performance would seem to be capacity limitations, given the requirement to keep track of current and past responses of multiple elements; a demand that would seemingly be imposed on working memory (see also Gao et al., 2019). Thus, the current experiment aims to offload some of the working memory limitations of the human operator through a visual aid, detailed below.

In sum, the collective wisdom of the studies described above reveals several features relevant to elements also present in the current ShadowHunt experiment:

1. The degrading effect of *set size* was strong and consistent when manipulated in previous MOT and visual search work, implicating several aspects of a serial visual search process (although see Gao et al., 2019, for a qualification). The set size manipulation provides one feature examined in the current experiments.
2. The *noise*, or unpredictability of movement has also been consistently found to degrade performance. Although not manipulated in the current experiment, the effect of noise was prominent in Patton et al. (2021) leading us to select a level here (25%) that produced a diagnostic accuracy of around 60%—well below perfect, even if it was much greater than the chance level of around 12%. Such an obtained level of accuracy is not uncommon in real world inference and prediction tasks in noisy and dynamic decision environments, such as with event predictions (Tetlock, 2017), financial predictions (Kahneman, 2011; Silver, 2012), or many types of medical diagnoses (Kahneman et al., 2021).
3. The role of *visual-spatial working memory* has been examined by several researchers and explicitly modeled by Gao et al. (2019). Indeed, working memory has been shown to be involved in multiple object tracking in two main ways: tracking and capacity. Spatial working memory capacity is directly involved in the ability to track multiple objects (Meyerhoff et al., 2017; Zhang et al., 2010). In the current study, tracking becomes more complex as participants must hold in their working memory the directional changes of their ship and all other ships on the screen to compare usership movements with a potential hostile ship’s movements. Therefore, increasing the number of potential target objects will increase the load on working memory, and even more so as more consecutive moves need to be remembered for comparison. As mentioned in the dynamic decision making literature, poor performance in these types of paradigms may be related to capacity limitations—in this case, a primary candidate would be working memory capacity limitations.

The potential role of spatial working memory capacity in inhibiting performance in the current paradigm led to the current investigation of how adding history trails, or “dot drops,” to the movement of all ships could improve detection performance by offloading memory requirements for past positions. This technique has been rarely examined, as most current aids are based upon artificial intelligence or highlighting (Riveiro et al., 2018; St. John et al., 2005; Vallières et al., 2016; see Onnasch et al., 2014 for a summary). History trails are a simple aid that displays a direct perception of a trajectory through displaying several dots from the past few display updates. This technique aligns with a fundamental principle of ecological interface design: replacement of memory by perception (Bennett & Flach, 2013; Burns et al., 2008).

History trails were previously an artifact of slow decay phosphorus blips on radar displays (Krishna, n.d.) and colloquially cited as useful. They were also used by the Navy to show past positions of ships (Frieden, 1978; pp. 20–21), but the extent of their benefit to performance was not quantified. Two previous studies of history trails in process control revealed no benefit to performance (West & Clark, 1974; Yin et al., 2015), however, performance in these studies was based on prediction rather than trajectory tracking and detection/diagnosis.

It is clear from previous research that the detection of hostile intention for movement is difficult. Cognitive limits such as working memory capacity and attention make keeping track of more than a few objects difficult. In applied settings, such as detecting a hostile ship before it attacks, it is important that the operator is accurate. Therefore, understanding what makes this task difficult is the first step in creating aids to increase performance. Working memory and set size are two variables that consistently appear throughout the relevant literature as limiting factors, and are investigated here.

### Current study

The current research investigates human abilities to detect hunting (closing in on a target) and shadowing (mimicking the movements of a target) behaviors in a simulated maritime environment. This environment captures prototypical features of Naval displays such as the Aegis (Smith et al., 2004). The task itself also mimics, in some ways, a real Naval task of a ship’s intelligence officer monitoring radar tracks, typically in a combat information center. Introducing variability into the ship movements can reflect natural variations of boats due to tides, weather, or other unexpected hazards. Additionally, smaller vessels that are able to move more quickly could easily add variability into their movements to mask their intentions. Deviations from normal paths and close approaches are both real ship movements that can indicate hostility (Lane et al., 2010).

Baseline performance data (Patton et al., 2021) indicate that detection performance is far below optimal and suggest that it may be due to working memory and attention limits. The current experiments aim to understand the extent to which these mechanisms impact detection performance. In the two experiments reported here, participants control their own ship (usership) in the open sea while other ships move around them. Within 35 discrete ship movements, participants must determine which ship is hostile and if it is exhibiting hunting or shadowing behaviors. Potential factors that may influence detection are manipulated in two ways. In Experiment 1, we examine the benefits of a “history trail” aid displayed for all ships to mitigate memory demands of keeping track of ship trajectories. In the second experiment, set size (number of distractor ships) and history trails are both manipulated orthogonally to determine the interaction between them and help understand the source of any benefits of the history trail. In both experiments, we look to replicate the distance by behavior interaction seen by Patton et al. (2021).

## Experiment 1

The ShadowHunt paradigm used in Patton et al., (2021) exerts a high cognitive load on working memory as participants track the current and previous locations of both their own ship and other distractor ships. Therefore, reducing working memory demand may increase accuracy in detecting the hostile ship. Although history trails have not been examined in this type of paradigm, it is expected that they should serve to offload the spatial working memory load of remembering the prior locations of all the items into perception. This should avail more cognitive resources for other aspects of detection of the hostile ship and therefore improve performance.

Patton et al. (2021) found a strong decline in detection accuracy when the hostile ship was hunting at a far distance. Current aids, based mainly on artificial intelligence and highlighting (St. John et al., 2005; see Riverio et al., 2018 for review), tend to fail at far distances (Dahlbom & Nordlund, 2013). Therefore, it is important to specifically examine the impact of history trails at far distances when evaluating their potential for performance enhancement.

Two hypotheses were proposed. First, it was hypothesized that history trails would increase overall detection accuracy of both hunting and shadowing because they will reduce the load on working memory and allow more cognitive resources to be used for detection. Second, as found in Patton et al. (2021), it was hypothesized that the detrimental effect of distance on detection and diagnosis would be larger for hunting than shadowing. Additionally, given prior work suggesting that distance effects can be important, we were interested in the impact of trails at far distances across both behaviors, although no directional hypothesis was posed.

### Methods

#### Participants

Each participant gave informed consent prior to commencing the experiment. Data were collected from 35 people on Prolific, all of whom were located in the United States. Two datasets were removed due to a combination of performance under chance, on average using less than eight of 35 possible steps, and further evidence of inattention from large time lags between interactions with the program.

#### Task

**Fig. 1:**
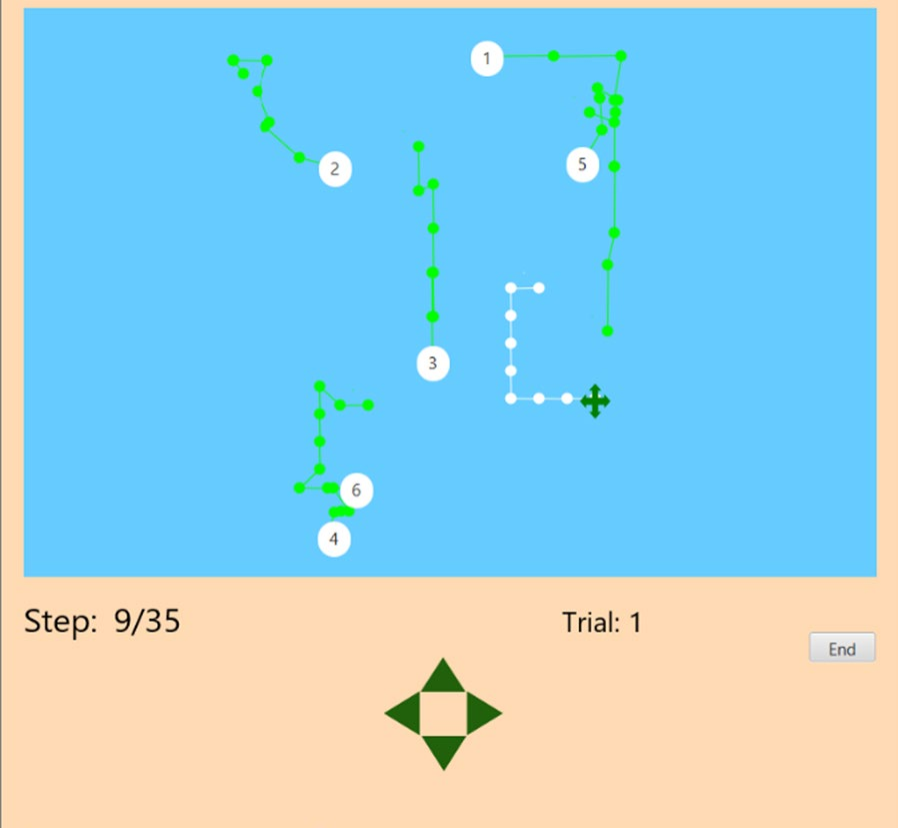
Screen exhibiting the experimental paradigm. Participants controlled the green cross while the white circles represent computer controlled ships. Green and white lines are history trails indicating the past position of each ship

Participants viewed a computer screen (see Fig. 1) containing a green cross indicating their ship’s position, which they could control, and six white circles with numbers which represented other ships and were controlled by a software application.

On each trial, the starting location of all ships was randomly generated. The participant’s ship could be moved in one of 4 directions (up, down, left or right) by clicking the arrow keys at the bottom of the screen. This movement produced a small jump by the usership in the chosen direction on the screen. These arrow keys could only be clicked once per second to negate the potential to create apparent motion through rapid keystrokes. There was no time limit on when the next movement had to be made. The movement of the participant’s ship on the screen was accompanied by an update of the computer-controlled ships, although these ships were able to move diagonally. Thus, all ships moved at the same time, with at least a one second delay in between movements.

On every trial, one of the computer-controlled ships was randomly selected to act in a hostile fashion. All ships were assigned a number for identification, thus making every ship a potential target. The hostile ship’s movements were contingent on the user’s movements. The hostile ship would do one of two things—hunt or shadow. Hunting meant moving in a way such that it would eventually reach the usership. An algorithm computed which directional movement produced the greatest reduction in distance between the usership and the hostile ship, and moved the hostile ship in that direction as the usership moved. Shadowing aimed to generally keep a consistent distance from the usership through replication of their movements. For instance, if the user moved left, the shadowing ship also moved left. If the usership moved toward the shadowing ship, it moved the same direction as the user so the distance between the ships stayed the same. These target movements occurred simultaneously with the usership movement that triggered it.

The other five ships on the display moved independently of the user’s actions. The behavior of the five non-hostile ships were randomly assigned other movement patterns. Three of the ships moved toward their own fixed target location, coded as an invisible point on the coordinate grid. The other two ships exhibited “patrol” behaviors, where they moved in a rectangular course that covered either 1/3, 1/2 or 2/3 of the screen. They could be oriented in any direction and the ship could start at any point on the path. A passive version of the task in which the viewer is not actively controlling ship movement can be accessed through the files at the link [https://osf.io/vkfdr/].

Movements of all computer-controlled ships contained 25% noise, such that, on average, every one out of four moves was not as expected for that ship’s programmed behavior. For example, if the hostile ship was shadowing, approximately every one out of four moves would not be the same as the usership. On half of the trials, all ships left history trails—dots indicating the ship’s previous nine positions, with lines connecting the dots. The usership’s trail was white, and all computer-controlled ships’ trails were green. The trials with history trails were blocked and randomized. Participants received four blocks of nine trials, two blocks with and two blocks without history trails.

Two initial practice trials demonstrated hunting and shadowing behaviors, with no data collected. Unlike in the experimental trials, on each practice trial the hostile ship was a different color and the hostile behavior was announced when the trial started. This allowed participants to practice working through a scenario but also showed the difference between hostile behaviors.

On each trial, the participant was required to make at least five moves, but no more than 35 moves, in whatever pattern they chose before determining which ship they believed was hostile. Once they made a decision, they clicked an “End” button. The ship display froze, and the participant indicated whether they were being hunted, shadowed, or neither. If they chose hunting or shadowing, the next question asked them to choose which ship was exhibiting that behavior by clicking the radio button that matched the ship number they believed was hostile. They then clicked “submit” and were given feedback only on the correctness of their response, but not on the correct target nor the hostile behavior exhibited on the trial. Ending the trial before 35 moves was at the discretion of the participant. Participants completed 36 trials (18 with and 18 without history trails), which took approximately 45 min.

### Results

Overall, participants correctly detected the hostile ship and behavior 62% of the time (chance performance for guessing both the correct ship and type of hostile behavior is 8.3%). Accuracy, operationalized as correct detection of both the hostile ship and its behavior, was the dependent variable for all analyses. Notably, when the correct ship was detected, the correct behavior was also detected 92% of the time. When the correct behavior was chosen, it was only assigned to the wrong ship 16% of the time and for the reverse, the wrong ship was assigned the correct behavior 38% of the time. This indicates that ship and behavior detections were closely coupled. Additionally, there was a significant (*t*(31) = −2.12, *p* = 0.003, *d* = 0.55) bias to report shadowing (73%) more than hunting (37%) on error trials.

**Fig. 2:**
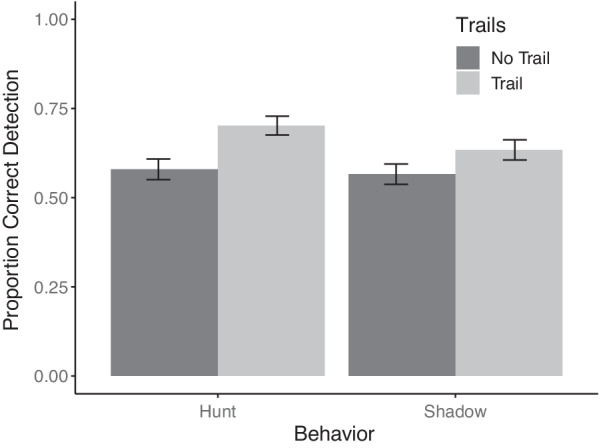
Percentage of correct detections by behavior with and without history trails. Error bars represent one standard error

Due to the randomization of starting distance across trials and the resulting large discrepancies in the number of trials at each distance that participants received, overall inferential statistics were not conducted across all three-way effects of distance with trails and behavior. A 2 (history trail) × 2 (behavior) repeated measures ANOVA was conducted. The proportion of correct trials for each behavior with and without trails was calculated for each participant. There was a main effect of history trail (Fig. 2), with small but significant benefits to performance on trials with history trails (66%) compared to those without (57%; *F*(1,32) = 8.15, *p* = 0.007, $\eta_{p}^{2}$ = 0.20). There was no significant main effect of behavior (*F*(1,32) = 1.52, *p* = 0.22, $\eta_{p}^{2}$ = 0.04), nor interaction between trails and behavior (*F*(1,32) = 0.81, *p* = 0.37, $\eta_{p}^{2}$ = 0.02) consistent with comparable performance benefits from history trails for both types of hostile intent.

**Fig. 3:**
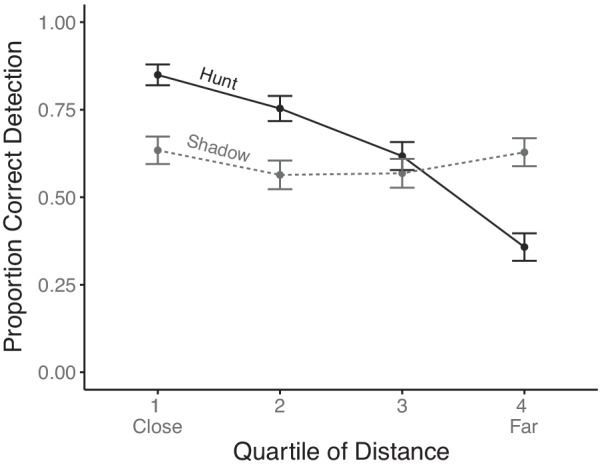
Detection accuracy as a function of behavior and starting distance between the usership and hostile ship, divided into quartiles. Error bars represent one standard error

Based on Patton et al.’s (2021) finding of highly degrading effect of increasing distance on hunting detection but not on shadowing detection, a planned examination of the impact of history trails under those circumstances was conducted. The 2 (behavior) × 4 (starting distance separation quartile) repeated measures ANOVA was conducted. As shown in Fig. 3, there was a main effect of distance (*F*(3,78) = 10.67, *p* < 0.001, $\eta_{p}^{2}$ = 0.29), with worse performance at further distances, and, as before, no main effect of behavior (*F*(1,26) = 1.68, *p* = 0.20, $\eta_{p}^{2}$ = 0.06). The interaction was significant (*F*(3,78) = 5.98, *p* < 0.001, $\eta_{p}^{2}$ = 0.18), indicating the minimal degrading effect of distance for shadowing (simple main effect: *F*(3,96) = 2.18, *p* = 0.09, $\eta_{p}^{2}$ = 0.06) compared to the large drop off with hunting (simple main effect: *F*(3,78) = 12.28, *p* < 0.0001, $\eta_{p}^{2}$ = 0.32), thus replicating the prior findings of Patton et al. (2021) and supporting the second hypothesis. Using accuracy for only those participants who encountered all four conditions at the longest distance, a 2 (trails) by 2 (behavior) repeated measures ANOVA produced no hint of a significant interaction (*F*(1,21) = 0.025, *p* = 0.87, $\eta_{p}^{2}$ = 0.001), but a significant main effect indicating the consistent benefit of trails (*F*(1,21) = 6.54, *p* = 0.01, $\eta_{p}^{2}$ = 0.23).

#### Speed accuracy trade-off

There was no difference in the mean number of steps used on trials with history trails (15.5) compared to trials without (15.8; *t*(34) = −0.51, *p* = 0.61, *d* = 0.03). The similarity in steps used combined with differences in accuracy indicates that history trails allowed people to accumulate more diagnostic evidence from the same number of steps. There was no difference in average time spent between steps (*M* = 2.0 s) with and without history trails.

We examined the speed-accuracy tradeoff between participants to assess the extent to which those who accumulated more evidence (more steps) also performed better. This examination revealed a positive correlation of *r* = 0.21 between average number of steps (per participant) and mean accuracy.

### Discussion

Our first hypothesis was that history trails would increase accuracy, which was confirmed, although the gains observed were rather modest. Specifically, history trails supported an overall improvement in detection (9%), including at further distances, which is important to note because current hostile intention detection aids tend to fail at far distances (Dahlbom & Nordlund, 2013). The findings are therefore consistent with history trails reducing working memory demands. Working memory (WM) is involved in the detection of hostile ships as movements had to be held in WM, then combined to form a trajectory, and then compared to the usership trajectory. We infer that WM decay and capacity limits impact the ability of an operator to hold all of the trajectories in memory, as revealed in other studies of and involving multi-object tracking (e.g., Gao et al., 2019; Harris et al., 2020). With the visual aid of the history trail, trajectories were able to be perceived, rather than remembered and imagined. This approach is congruent with the concept from ecological interface design that replacing memory with perception improves performance (Bennett & Flach, 2013), as well as the idea that a “visual echo” can offset vulnerabilities of working memory (Helleberg & Wickens, 2003). The offloading of trajectories also allowed detection accuracy to improve without a change in the number of steps or evidence accumulated.

However, the size of the benefit derived from the addition of history trails was fairly modest, given that even with trails, accuracy was still only 66%. Thus, the limits on performance are either not purely resulting from working memory, or the remaining demands on working memory in the performance of the task continue to overwhelm its limited capacity even with history trails supporting certain aspects. We return to this issue in Experiment 2.

The second hypothesis, that there would be degrading effect of distance on hunting but not shadowing (seen in Patton et al., 2021) was confirmed. The clear decrement to detection of hunting at a distance indicates that something about hunting behavior is qualitatively different than shadowing for the perception of patterns. This could be because a shadowing hostile ship and usership can be treated as if they are connected by a virtual semi-rigid line, which has been shown to improve tracking performance (Yantis, 1992; see Patton et al., 2021 for a further discussion).

## Experiment 2

Experiment 1 showed that history trails improved performance, implicating a role for reducing working memory load. However, a detrimental effect of distance was present with history trails and overall detection accuracy; even with history trails, performance was 66%. This indicates that there may still be an overload on working memory capacity, even with trails. To investigate this possibility, Experiment 2 used the same methodology, this time with five and two distractors. If it is the case that working memory is still overloaded with history trails, performance should be improved when the number of distractors is decreased.

Two main hypotheses were posed: First, that the effects of Experiment 1 will replicate. Second, performance will be better with two distractors than five because the load on working memory of keeping track of their behavior will be less.

### Methods

#### Participants

This research complied with the American Psychological Association Code of Ethics and was approved by the Institutional Review Board at Colorado State University. Each participant gave informed consent prior to beginning the experiment. Data were collected from 33 people on Prolific, all of whom were located in the United States.

#### Task

The task was the same as Experiment 1, except as noted here. Participants completed 36 trials in four blocks of nine trials. Each block had either three (the target plus two distractors) or six computer-controlled ships (the target plus five distractors). Half of the blocks contained history trails and the other half did not. This created four conditions: three or six ships, with or without history trails. The blocks were presented in a random order for each participant.

### Results

Overall, participants correctly detected the hostile ship and behavior 53% of the time. Notably, when the correct ship was detected, the correct behavior was also detected 86% of the time. When the correct behavior was chosen, it was only assigned to the wrong ship 22% of the time and when the wrong ship was chosen, the correct behavior was chosen 39% of the time. This again indicates, as in Experiment 1, that ship and behavior detections were closely coupled. Additionally, there was a non-significant (*t*(32) = −0.64, *p* = 0.52, *d* = 0.11) bias to report shadowing (54%) more than hunting (46%) on error trials.

Accuracy above chance was the dependent variable for all analyses. Accuracy was operationalized as correct detection of both the hostile ship and its behavior, and the level of chance accuracy (16% for set size 3, 8% for set size 6) was subtracted from the raw accuracy score for all analyses so that the natural increase in performance with a lower set size would not be a confound.

**Fig. 4:**
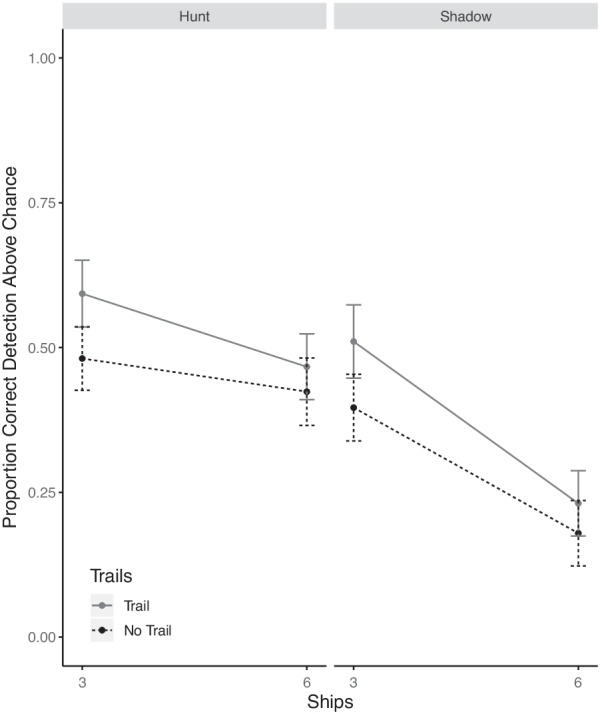
Detection accuracy above chance as a function of set size, history trails and behavior. Error bars represent one standard error

Figure 4 presents the joint effects of set size, behavior, and history trails on accuracy. A 2 (set size) by 2 (behavior) by 2 (trail) repeated measures ANOVA revealed a main effect of distractors (*F*(1,32) = 32.6, *p* < 0.001, $\eta_{p}^{2}$ = 0.50) with better performance for smaller set sizes, a main effect of history trails (*F*(1,32) = 7.44, *p* = 0.01, $\eta_{p}^{2}$ = 0.18) with better performance when history trails were present, and a main effect of behavior (*F*(1,32) = 13.68, *p* < 0.001, $\eta_{p}^{2}$ = 0.29) with superior identification of hostile intent when the behavior involved hunting. There was a significant interaction between distractors and behavior (*F*(1,32) = 7.22, *p* = 0.01, $\eta_{p}^{2}$ = 0.18), which can be seen in the much larger cost of increasing distractors for shadowing than for hunting with six ships (simple main effect: *F*(1,32) = 20.99, *p* < 0.001, $\eta_{p}^{2}$ = 0.39) as compared to three ships (simple main effect: *F*(1,32) = 2.52, *p* = 0.12, $\eta_{p}^{2}$ = 0.07). No other interactions were significant (all *p* > 0.10). The findings are therefore consistent with benefits of trails being similarly realized across both set sizes and both behaviors.

**Fig. 5:**
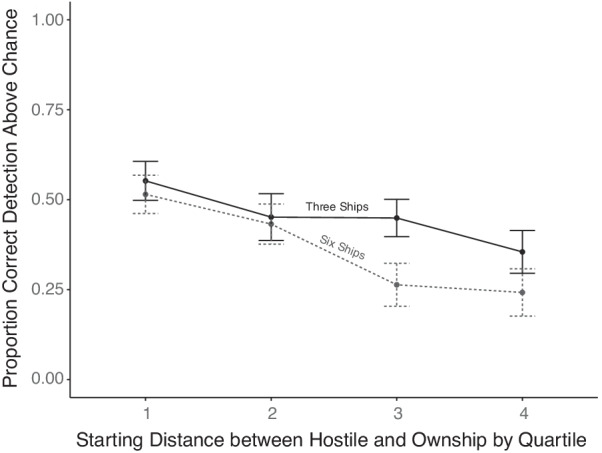
Detection accuracy for each set size as a function of the starting distance between the usership and hostile ship. Error bars represent one standard error

The same issues for an analysis of distance effects in Experiment 1 were also present in this experiment. In particular, the uneven distribution of observations across participants and distances again created a very small sample at some distances for specific combinations of history trails and distractor numbers, so it was impossible to run a fully factorial ANOVA. In the following, we only examined and report 2-way interactions between distance, and each of the other three variables in turn. Figure 5 presents the effects of distractors and distance.

A 2 (set size) by 4 (distance) repeated measures ANOVA revealed a main effect of distance (*F*(3,69) = 7.93, *p* < 0.001, $\eta_{p}^{2}$ = 0.25) with worse performance at further distances. Replicating the effects of the analysis of the data in Fig. 4, there was also a main effect of distractors (*F*(1,23) = 8.44, *p* = 0.007, $\eta_{p}^{2}$ = 0.26) where accuracy was higher with fewer distractors. The interaction was significant (*F*(3,69) = 3.13, p = 0.03, $\eta_{p}^{2}$ = 0.11) and can be seen in the larger cost of increasing distance with six ships (simple main effect: *F*(3,78) = 7.40, *p* < 0.001, $\eta_{p}^{2}$ = 0.22) than with three ships (simple main effect: *F*(3,84) = 4.42, *p* = 0.006, $\eta_{p}^{2}$ = 0.13).

A 2 (trail) by 4 (distance) repeated measures ANOVA revealed, as before, a main effect for a benefit of trails (*F*(1,27) = 5.16, *p* = 0.03, $\eta_{p}^{2}$ = 0.15) and a main effect of distance (*F*(3,81) = 4.28, *p* = 0.007, $\eta_{p}^{2}$ = 0.13). The interaction between trails and distance was not significant (*F*(3,81) = 0.50, *p* = 0.68, $\eta_{p}^{2}$ = 0.01).

**Fig. 6:**
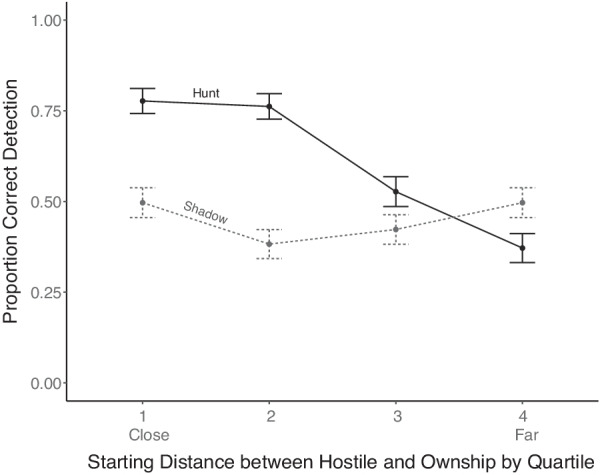
Joint effects of distance and behavior type on detection accuracy. Error bars represent one standard error

A 2 (behavior) × 4 (distance) repeated measures ANOVA was conducted on the data presented in Fig. 6. As in previous experiments, there was a main effect of distance (*F*(3,69) = 8.83, *p* < 0.001, $\eta_{p}^{2}$ = 0.27) with lower accuracy at further distances. The analysis again revealed a main effect of behavior (*F*(1,23) = 8.58, *p* = 0.007, $\eta_{p}^{2}$ = 0.27) where hunting detection was generally superior to shadowing. The interaction between behavior and distance was again significant (*F*(3,69) = 9.84, *p* < 0.001, $\eta_{p}^{2}$ = 0.30). This can be seen in the large degrading effect of distance on hunting (simple main effect: *F*(3,75) = 17.75, *p* < 0.001, $\eta_{p}^{2}$ = 0.41), which was entirely absent for shadowing (simple main effect: *F*(3,84) = 0.86, *p* = 0.46, $\eta_{p}^{2}$ = 0.03), mirroring the effects in both Experiment 1 (see Fig. 3) and Patton et al. (2021).

#### Speed Accuracy Trade Off

The average number of steps taken overall was 14.95. There was no difference in the average number of steps taken between trials with history trails (14.93) and those without trails (14.97). There were, however, fewer steps used with two distractors (*M* = 13.96) than with five distractors (*M* = 15.94; *t*(32) = −3.25, *p* = 0.002, *d* = 0.29).

We also examined the speed-accuracy tradeoff between participants, to assess the extent to which those who accumulated more evidence (more steps) also performed better. This examination revealed a small correlation between average number of steps (per participant) and mean accuracy, of *r* = 0.12.

### Discussion

Supporting the first hypothesis and replicating the effects seen in Experiment 1, history trails increased overall accuracy, accuracy decreased with distance, and hunting detection suffered from increasing distance more than shadowing. This interaction between distance and behavior is consistent across all experiments (including Patton et al., 2021) and is important to note because it may suggest that detection of hostile intent is dependent on the behavior itself—and thus, it may be important in future work to identify the various behaviors that need the most support for accurate detection.

Also replicated from Experiment 1 and Patton et al. (2021) was the interaction between behavior and distance suggesting, as we describe in more detail in Patton et al. (2021), that a particular benefit for shadowing detection offsets the cost of greater separation. It may be that this is a result of the more rigid object-like properties conferred on a shadowing target connected by a virtual line of constant angle and orientation, which avails processing mechanisms of object-based attention (Yantis, 1992), although based on the current data, we cannot determine with certainty why this occurs.

For the second hypothesis, accuracy was seen to decrease with more distractors. This implies again that working memory plays an important role in the detection of hostile ships, as fewer distractors lends itself to a lower working memory load. The interaction between distance and distractors suggests that much of the detrimental effect of distance is a result of increasing distractors intervening between the hostile ship and usership, a factor both impeding visual search and the working memory demands of keeping track of candidate hostile ships, and diagnosing hostile behavior. The common resource demands of search and spatial working memory is well established (Liu & Wickens, 1990; Wickens, 2008).

## General discussion

The current experiments were designed to extend the line of research from Patton et al. (2021) and investigate how a visual aid and set size can impact the detection of hostile intent. In Patton et al. (2021), accuracy for detecting a hostile ship by movement alone was challenging, and the current experiments revealed that using history trails as a visual detection and diagnostic aid and fewer distractors both increased accuracy. Five major findings were present.

First, accuracy of detection increased as set size decreased. In a relevant paper with a similar paradigm, Gao et al. (2019) suggest that a linear accuracy effect with set size may reflect an interaction between a parallel pre-attentive search and a serial search for the target. We do not currently have the data to understand how participants searched for the hostile ship, but this idea of parallel search techniques supports the idea that most participants find the hostile ship by initially watching all objects on the screen and narrowing their focus as evidence accumulates. It is interesting that the loss of accuracy produced by increasing set size from 3 to 6 here (58–47%, respectively) was actually smaller than the corresponding accuracy loss from 3 to 6 observed by Gao and colleagues (90–50%), suggesting here the possibility of some parallel processing between search and diagnosis.

The impact of set size on accuracy, while not surprising, has important implications for real world hostile detection. The maximum set size here was only six, which is very small compared to the number possible on real ship radar screens, which can host hundreds of ships at a time. When detection rates are low with three and six ships, it follows that detection rates with dozens or hundreds of ships would be even lower, if the hostile ship was detected at all. This points to a vital need for decision support tools because humans acting alone under such high loads would be prone to missing movements that might signal hostile intent. Although set size within the waterways cannot be changed, there would be many options for radar displays to reduce set size such as lowlighting ships that have been determined non-hostile by an algorithm, or reducing the size of the visible waterway on the radar screen such that fewer ships are in the picture, equivalent to a technique employed in air traffic control of adapting the size of a sector for which an individual controller is responsible (Wickens et al., 1997). These are easy to implement options that should be considered in applied settings.

Second, in both experiments, history trails consistently improved diagnostic performance—a finding in partial contrast with previous work that found that history trails did not improve performance on tasks involving prediction of trends (West & Clark, 1974; Yin et al., 2015). However, prediction is substantially different from detection and diagnosis, so the failure to replicate these prior null results is not surprising. Here, we assumed that trails allowed offloading of the previous trajectories of ships from working memory to the display, thereby reducing the overall cognitive load of the task. Such offloading would allow more cognitive resources to be dedicated to processing ship movements and diagnosing the behavior of the hostile ship, thereby improving performance.

The lack of interaction between set size and history trails is particularly interesting. This may suggest that the primary benefit of history trails is in the diagnostic stage of choosing between hunting and shadowing, whereas the primary cost of increasing set size lies in an earlier sequential phase of the task, by which ships are examined in a serial search to identify which might be a likely candidate for the hostile target.

The third main finding from the two experiments was the influence of the two different kinds of hostile behavior. The differences in detectability of the two were inconsistent, with no differences in detection performance between shadowing and hunting observed in Experiment 1, nor in Patton et al. (2021), but a large difference in Experiment 2, where shadowing was detected more poorly than hunting. It is unclear why the significant difference exists here, although the combination of lower load plus trails might have induced strategies that did not generalize well to the other conditions, but this single finding should be interpreted with some caution.

Fourth, while the main effect of behavior type is inconsistent across experiments, one of the most consistent findings across both experiments here and in Patton et al. (2021) is the much greater detrimental effect of distance on detection of hunting, than on detection of shadowing. The source of the differential distance effect is likely due to the nature of hunting behavior. When a hunting ship is at a far distance, the movements it must make to approach the usership require less drastic changes in heading, making the movements less conspicuous. When a hunting ship is closer to the usership, the movements of the hunting ship typically require larger heading changes and become more obviously different from the movement of the non-hostile ships that took a straight trajectory. In contrast, shadowing movements are the same regardless of distance. Although this confounding variable is unable to be avoided in the hunting behavior, it is important to note that this is a real world issue faced by Naval operators, such as in the U.S.S Stark incident, where the initiation of a hunting-like behavior was not immediately detected by the combat information center (Smith et al., 2004).

Finally, regarding the strategy of the stopping point in evidence accumulation, we found some evidence that those who accumulated more performed better, a slight contrast with the results of Patton et al. (2021) who found a stronger correlation between steps and accuracy in a similar condition (*r* = 0.40, compared to *r* = 0.21 and *r* = 0.12, here).

### Limitations

The current paradigm is focused on basic research with real world applications, which inevitably lends itself to a few main limitations. The generalizability of the current paradigm and its results is limited by both the use of naïve participants, unfamiliar with maritime scenarios such as the ones presented here, and the simplicity of the current paradigm. However, these consequences of laboratory research allowed strong control over variables in order to examine the basic relationship of working memory load on detection of hostile intent. Additionally, the use of only two behaviors limits the ability to generalize the results of these experiments to other hostile detection scenarios. Aids for inferring other hostile spatial behaviors besides shadowing and hunting may need to be developed independently.

## Conclusions

The current research suggests that detection of hostile ships from movement alone is difficult, no matter the circumstances. Working memory limits likely play a role in the difficulty, as do the visual attentional limitations when potential targets are quite distant from usership on the screen. Most importantly, an easy to implement decision support tool of history trails improved performance in the current paradigm and would serve as a beneficial aid for subgroups of potentially hostile ships on a radar screen. Although other variables, such as clutter, still need to be investigated, the current studies provide a reasonable starting point for improving detection of hostile ships from spatial movements alone.

## Data Availability

The datasets used and/or analyzed during the current study are available from the corresponding author on reasonable request.
